# In Vivo Imaging of Microglial Calcium Signaling in Brain Inflammation and Injury

**DOI:** 10.3390/ijms18112366

**Published:** 2017-11-08

**Authors:** Petr Tvrdik, M. Yashar S. Kalani

**Affiliations:** 1 Department of Neurosurgery, University of Utah School of Medicine, Salt Lake City, UT 84132, USA; 2 Department of Neurosurgery, University of Virginia School of Medicine, Charlottesville, VA 22908, USA; kalani@virginia.edu

**Keywords:** microglia, calcium signaling, in vivo imaging, two-photon, GECI, PC::G5-tdT, inflammation, Alzheimer’s disease, ischemic stroke

## Abstract

Microglia, the innate immune sentinels of the central nervous system, are the most dynamic cells in the brain parenchyma. They are the first responders to insult and mediate neuroinflammation. Following cellular damage, microglia extend their processes towards the lesion, modify their morphology, release cytokines and other mediators, and eventually migrate towards the damaged area and remove cellular debris by phagocytosis. Intracellular Ca^2+^ signaling plays important roles in many of these functions. However, Ca^2+^ in microglia has not been systematically studied in vivo. Here we review recent findings using genetically encoded Ca^2+^ indicators and two-photon imaging, which have enabled new insights into Ca^2+^ dynamics and signaling pathways in large populations of microglia in vivo. These new approaches will help to evaluate pre-clinical interventions and immunomodulation for pathological brain conditions such as stroke and neurodegenerative diseases.

## 1. Introduction

Microglia are the principal innate immune cells of the brain parenchyma. Their distinctive developmental origin differentiates them from other myeloid cells of the central nervous system. Since Pio del Rio-Hortega systematically characterized microglial appearance and distribution [1], their mesodermal origin has been generally accepted. However, traditional belief maintains that microglia are descendants of circulating monocytes originating from bone marrow. This concept was first formally questioned by Alliot et al., who demonstrated that the origin of microglia can be traced developmentally to primitive hematopoiesis in the yolk sac [2]. More recently, new genetic tools have been developed in mouse models using gene targeting. Manipulation of loci specifically expressed in primitive hematopoietic cells, such as tagging the *Runx1* gene with CreER recombinase, allowed fate-mapping of primitive hematopoietic stem cells from the yolk sac. Precise timing of reporter labeling was enabled by inducing CreER-mediated recombination with injections of a selective estrogen receptor (ER) modulator, 4-hydroxytamoxifen [3]. These experiments provided unequivocal evidence that resident microglia in the brain are born in the yolk sac from primitive myeloid progenitors before embryonic day eight and populate the neural tube soon after [4]. Strong evidence for relatively independent postnatal development of cortical microglia was gained from parabiotic experiments, in which normal and GFP-labeled mice were surgically conjoined to share the same blood circulation. Among peripheral myeloid cells, 30% of the monocytes and tissue macrophages were GFP donor-derived after one month of parabiosis. However, less than 5% of microglia in parabiotic animals were found to be donor-derived [4]. These findings were corroborated by early neonatal bone marrow transplantations which yielded similar outcomes; although 30% of circulating leukocytes and tissue macrophages were of donor origin, 95% of adult microglia remained of host origin [4]. In support of these findings, Saederup et al. showed that bone marrow-derived RFP-labeled monocytes make little or no contribution to the parenchymal microglia in healthy animals [5]. Thus, the primitive microglial population can be self-renewing within the immune-privileged parenchyma throughout life, with a very slow turnover. In fact, human microglia can persist for more than two decades in the brain [6]. However, when the blood-brain barrier is compromised due to infection or injury, an influx of monocytes can occur from the peripheral myeloid population, giving rise to microglia that appear distinct from the primitive population [7]. Varvel et al. also showed that reduction of the resident microglial population with a conditional ablation system (herpes virus thymidine kinase and the pro-toxin ganciclovir) can facilitate microglial repopulation by monocyte infiltration [7]. In contrast, another study achieved microglial depletion with selective inhibitors of the colony stimulating factor 1 receptor (Csf1r) pathway which is necessary for microglial survival. In this model, the blood-brain barrier remains intact and the microglial population is replenished from putative stem-like microglia precursors within in the brain, not from circulating monocytes [8]. The complexity of the developmental origins of microglia has significance for aging and neurodegenerative disorders such as Alzheimer’s disease. It has been shown, for example, that different microglial populations originating from the primitive and bone marrow-derived pools can have different impacts on plaque formation [9].

During early postnatal development, microglia acquire ramified morphology with small cell bodies and elongated processes covering an area of approximately 50 µm in diameter in both mouse and human brains [10]. Davalos, along with others, discovered that differentiated microglial processes are constantly in motion [11,12]. They measured the turnover of protrusions and retractions of the processes and found velocities of up to 4.1 mm/min [12]. Although seemingly random in direction, microglial processes have been shown to protrude towards, and make connections with, neuronal synapses. Remarkably, the frequency and duration of these connections appear to be related to basal neuronal activity [13,14]. Following targeted injury, such as disruption of the blood-brain barrier or laser injury, the multidirectional movement of the microglial processes is switched to movement directed toward the injured site at similar speeds [11,15,16]. This level of activity qualifies microglia as the most motile cells within the brain parenchyma. Microglial surveillance behavior is energetically costly and deeper mechanistic insight into this intriguing phenomenon is still lacking. Nonetheless, extracellular nucleotides appear to be involved in regulating baseline motility and ramification because microglial scouting behavior is lost after infusion of apyrase, an ATP/ADP-degrading enzyme [11,17]. The principal receptors controlling the directional response are purinergic P2 receptors, primarily P2ry12, a Gi-coupled metabotropic P2 receptor which is highly expressed on microglial processes. This signaling pathway eventually leads to activation of protein kinase B (PKB) and reorganization of the actin cytoskeleton, leading to cell growth and chemotaxis [18]. The complete set of microglial signal receptors, termed the ‘sensome’, has been determined by direct sequencing of the transcriptome [19]. Gene ontology analysis has revealed that purinergic receptors account for 8% of the sensome. Other receptor groups include chemokine receptors (10%), cytokine receptors (10%), receptors for extracellular matrix proteins (6%), receptors involved in cell-cell interaction (10%), Fc receptors (7%), and other less-well characterized and orphan receptors (24%). A substantial part of the sensome is accounted for by pattern recognition receptors (25%), such as the Toll-like receptors (TLRs) which recognize conserved motifs of pathogen-associated molecular patterns (PAMPs) or damage-associated molecular patterns (DAMPs), molecular fragments released from necrotic or dying cells. Stimulation of TLRs causes induction of AP-1 and NF-κB and subsequent activation of multiple pro-inflammatory genes, as well as profound changes in morphology and migratory behavior [20].

The malleable properties of microglia present investigators with significant challenges. First of all, studies of ramified microglia are impeded by any damage to the meninges and blood-brain barrier which leads to rapid activation of the sensome. The distinct developmental origin of microglia complicates genetic manipulation of these cells, and commonly used techniques such as in utero electroporation or infection with viral vectors, are ineffective. The phagocytic nature of these cells also compromises the expression and affects the turnover of indicator dyes and reporters, such as microbial β-galactosidases. Above all, two technologies have spurred the advancement of microglial research in vivo, namely genetic manipulations of the mouse genome and two-photon in vivo imaging. Two-photon laser scanning fluorescence microscopy, pioneered by Winfried Denk and others [21], uses tunable femtosecond infrared laser pulses (~700–1100 nm) concentrated into a tiny focal volume (~1 cubic micron) with appropriate lenses and scanning galvanometers [22]. At this high density, infrared photons combine to excite commonly used green and red fluorescent proteins [23] and emit visible photons that can be efficiently detected using non-descanned photomultiplier tubes (PMTs) deep in the brain [22]. Microglial reporters can be then imaged several hundred microns deep in the cortex through the thinned-skull method or sealed craniotomy preparations [24]. Genetic knock-in technology complements the two-photon technique by providing the capability to specifically express genetically encoded fluorophores in the cells of interest [25]. Very instrumental in this regard was the generation of the *Cx3cr1*-GFP allele which enabled in vivo characterization of microglial behavior and motility [26], opening avenues for further investigation.

## 2. Early Exploration of Ca^2+^ Regulation in Cultured Microglia

Intracellular Ca^2+^ acts as a second messenger in virtually all cell types, including immune cells [27], and many microglial responses are thought to be mediated by intracellular Ca^2+^ signals [28]. Until recently, the bulk of information on microglial Ca^2+^ signaling has been obtained from cultured cells. The effort began in the early 1990’s by the groups of Carl Cotman and Helmut Kettenmann [29,30]. Since cell culture approaches offer good access and effective loading with synthetic Ca^2+^-indicating probes, such as Fura-2, this strategy yielded information on a number of pathways, including purinergic and complement signaling [31,32,33,34,35]. However, experiments involving cultured microglia have many caveats. First, microglia cannot be isolated in their resting state, and obtaining differentiated ramified morphology in culture is challenging. Secondly, the most common culturing procedures use dissociated cells from early neonatal brains, in which microglia have not yet matured. Therefore, much of the early literature stems from studies on cell cultures that were in an activated state and do not represent the resting microglia in the healthy brain. Even in acute brain slices, despite some evidence to the contrary [36], microglia become activated and their Ca^2+^ levels are elevated [37]. Also, radical differences between in vitro cultured primary microglia and microglia isolated immediately ex vivo were found by gene expression analysis [38,39]. Thus, it has become evident that investigating Ca^2+^ signaling in vivo is necessary for accurate understanding of microglial behavior in the normal and diseased brain.

## 3. Imaging Microglial Ca^2+^ Activity In Vivo

### 3.1. Strategies Employing Cell Electroporation 

Microglia have been refractory to initial attempts at synthetic dye loading and viral transduction in vivo. Taking an innovative approach, Eichhof et al. adapted a previously developed single-cell electroporation method [40] and labeled microglia in vivo by mild electroporation [41]. This opened the possibility of visualizing microglial Ca^2+^ in vivo for the first time. The authors targeted microglial cells, labeled with isolectin B4, with an electroporation pipette filled with 10 mM Oregon green BAPTA 1 (OGB-1), and applied negative current for 10 ms. This technique allowed labeling of both microglial somas and processes, and enabled high-resolution two-photon imaging in acute in vivo experiments. This study demonstrated that the majority of ramified, unchallenged microglia display no spontaneous Ca^2+^ transients. However, single-cell damage within a radius of 50 µm caused large Ca^2+^ transients, which were dependent on purinergic receptor (P2Y) signaling and intracellular Ca^2+^ stores [41]. This approach offers flexibility of indicator choices and the ability to target select cells, but it is laborious and not applicable to longitudinal studies in large cell populations.

### 3.2. Viral Strategies for Transducing Microglia 

Although partial success in delivering adeno-associated virus (AAV)-encoded gene expression to microglia has been reported for AAV-5 serotypes and transgenes expressed from F4/80 (*Adgre1*) promoters, its specificity in vivo has not been critically evaluated [42]. In neurons and astrocytes, a frequently used AAV strategy employs mouse strains expressing Cre recombinase in the cells of interest and subsequent infection with Cre-dependent, flip-excision (FLEX) configured AAV vectors. As a result, conditional AAV-mediated gene expression in the Cre-expressing cells is achieved [43]. No published data is available on intracerebral transduction efficiency of the AAV-FLEX approach in microglia, but the initial results are encouraging [44].

In the first report of virally-delivered, genetically encoded Ca^2+^ indicators (GECIs), Seifert et al. resorted to labeling microglia with a retrovirus [45]. Since retroviruses only infect dividing cells, the authors used stab wound injury to trigger microglial proliferation. Consequently, this experimental paradigm resulted in targeting a heterogeneous population of microglia and macrophages which could not be differentiated with tomato lectin staining. Nevertheless, this was the first study in which a GECI was delivered to microglia in vivo. The indicator used was a single-wavelength GCaMP2 [46], consisting of a circularly permuted GFP (cpGFP), calmodulin, and the M13 fragment from myosin light chain kinase [47]. Fluorescence intensity of the circularly permuted GFP is modulated by Ca^2+^ binding-induced structural changes in the chromophore. Solving the crystal structure of GCaMP2 [48,49] led to rationally and empirically guided improvements of indicator properties in GCaMP3, featuring greater protein stability, a larger dynamic range, and higher affinity for Ca^2+^. GCaMP3 was the first universally applicable GCaMP reporter for in vivo studies in a variety of cell types, in organisms ranging from worms to flies and mice [50]. Although it has never been reported in microglial investigations, we used GCaMP3 with good success in rat astrocytes following in utero electroporations [51].

Brawek et al. took a different approach for gene expression delivery to microglia [37]. To ensure specific expression in microglia, they used a lentivirus vector destabilized by including target sites for microRNA-9 (miR-9), which mediate degradation of the tagged mRNA in cells expressing the complementary miR-9. In contrast to most brain cells, microglia lack miR-9 expression, and brain infection with miR-9-regulated lentivirus results in selective labeling of microglial cells [52]. Brawek and colleagues chose to express a new fluorescence resonance energy transfer (FRET)-based Ca^2+^ biosensor Twitch-2B [53], comprising mCerulean3 and cpVenus^CD^ as a FRET donor and acceptor [54]. The fluorescent proteins are connected through a high-affinity Ca^2+^ binding linker from troponin C isolated from the toadfish. This molecular configuration lends a maximal fluorescence ratio change of ~800% and a high Ca^2+^ affinity K_d_ of 200 nM, but a relatively slow decay time of 2.8 s [53]. The ratiometric FRET imaging is less influenced by excitation laser intensity, indicator expression level, or by changes in the optical path length, and therefore, it is better suited to detect sustained elevations in Ca^2+^ levels. This study confirmed that microglia have very little spontaneous activity under steady-state conditions. In contrast, ratiometric measurements revealed that in vivo steady-state Ca^2+^ levels in microglia are actually higher than those in neurons, suggesting that microglia have a different set-point of cytosolic Ca^2+^. In cell cultures or slice preparations, ramified microglial cells consistently showed significantly higher and very heterogeneous Ca^2+^ levels [37]. These findings reveal that ramified microglia often sustain elevated intracellular Ca^2+^ levels in response to changes in the environment. However, the miR-9-based viral approach was not consistently cell-type specific. When viral expression was high, it labeled other cell types. At lower concentrations, it yielded only spotty microglial labeling, precluding systematic surveys of microglial populations. While the ratiometric approach is promising, it suffers from inevitable photon loss due to emission splitting, and requires a specialized configuration of PMT detectors.

### 3.3. Endogenous Reporter Systems for Microglial Imaging 

Genetically encoded Ca^2+^ indicators have been improved to the extent that their properties match or exceed those of synthetic indicators [55,56]. Unfortunately, the development of transgenic models expressing these genetic tools in microglia has been impeded by the discrepancies of microglial gene expression. A case in point is the effectiveness of the *ROSA26* locus. This gene displayed ubiquitous expression in all cells of the embryo [57,58] and this observation established *ROSA26* as a widely used insertion site for endogenous reporters and effectors. Although the locus supports expression in microglia, the expression levels are not nearly as high as in other brain cells. Consequently, Ca^2+^ sensors expressed from *ROSA26*, such as the GCaMP3 indicator, did not perform well in microglia, although it robustly labeled neuronal populations in the retina, cortex, and cerebellum [59].

**Figure 1:**
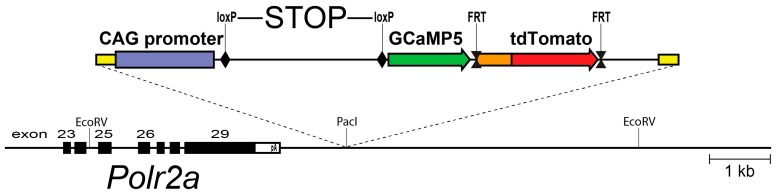
A schematic diagram of the PC::G5-tdT reporter allele in the *Polr2a* locus. The reporter cassette was inserted 3′ of the last exon, without disturbing gene function. Following Cre/loxP-mediated excision of the transcriptional STOP sequence, the CAG promoter drives GCaMP5G and IRES-tdTomato expression in the Cre-expressing cells (e.g., *Aif1*(Iba1)-IRES-Cre directs expression to the myeloid-monocytic lineage). If red fluorescence is not desirable, the IRES-tdTomato reporter can be independently removed with FLP recombinase using the FRT sites. Modified from [60].

Using an alternative strategy, we explored the genomic context of the largest RNA polymerase II subunit, the *Polr2a* gene. This gene is essential and ubiquitously expressed, and we reasoned that this locus could support efficient expression in neurons as well as glia. A Cre-dependent expression cassette, driven by the strong CAG promoter, was inserted 3′ adjacent to *Polr2a*, without disturbing the function of the gene. This arrangement allows efficient breeding of homozygous animals. After Cre recombination, the reporter (named PC::G5-tdT) expresses the single-wavelength Ca^2+^ indicator, GCaMP5G, and the red fluorescent protein tdTomato (Figure 1). This reporter proved to support strong expression of a wide array of cell types and systems, including microglia and astrocytes [60].

To express the reporter in all microglia, we generated a new Cre driver by inserting the IRES-Cre cassette in the 3′ untranslated region of the *Aif1* gene, encoding the allograft inflammatory factor 1, also known as ionized Ca^2+^-binding adapter molecule 1 (Iba1) [16]. Simultaneous expression of tdTomato in the target cells is very advantageous because basal fluorescence of the new generations of GCaMP indicators, including GCaMP5G, is low and does not enable visualization of cell bodies and processes in the off-state. However, this permits GCaMP5G’s great dynamic range of 1085 ± 66%. This indicator has a rapid decay time of 667 ± 43 ms and an intermediate Ca^2+^ affinity with K_d_ = 447 ± 10 nM [55,61]. Efficient excitation of both markers is achieved with infrared lasers tuned to 920 nm and detected with common filters for GFP and Tomato emission. 

Our genetic system has enabled, for the first time, in vivo imaging of Ca^2+^ activity in the entire microglial network. The findings obtained with endogenous genetic indicators converge with findings obtained with other approaches, including synthetic dyes, showing that spontaneous Ca^2+^ transients are very infrequent [16,41]. In our experiments, only 4% of resting microglia exhibited at least one spontaneous Ca^2+^ transient during a 20-min recording session [16]. This is in stark contrast to the remarkable motility of microglial processes, implying that the constant motion in the unchallenged, ‘resting’ state occurs in the absence of detectable Ca^2+^ fluctuations. Our in vivo results with BAPTA-AM, an intracellular Ca^2+^ chelator, have indicated that basal levels of Ca^2+^ are necessary for full protrusion motility [16]. However, these transients may occur in microdomains similar to the phenomena observed in astrocytes [62] and/or might be too low to be detected with existing technology.

## 4. Patterns of Microglial Ca^2+^ Activity in Inflammation 

### 4.1. Microglia Play a Central Role in Brain Inflammation 

The innate immunological responses of the nervous system are usually referred to as neuroinflammation. These processes primarily involve microglia and astrocytes, leading to pathological states of microgliosis and astrogliosis, which are characterized by pronounced changes in gene expression, cellular structure, and function [63,64]. In contrast to classical peripheral inflammation, mobile immune cells including macrophages, monocytes, neutrophilic granulocytes, and lymphocytes, are not always recruited to the inflamed site, subject to blood-brain barrier integrity [65]. Conversely, peripheral cytokines may access the brain with an intact blood-brain barrier by passive diffusion through the choroid plexus or circumventricular organs [66] by active transport through the brain endothelium [67], or by the activation of vagal, trigeminal, or glossopharyngeal afferent fibers to indirectly promote cytokine production by astrocytes and other cells within the CNS [68,69,70]. 

Another specific of nervous system inflammation is the higher prevalence of sterile inflammation, typically due to trauma or ischemia-reperfusion injury rather than direct infection with microorganisms [71,72]. Sterile inflammation can be triggered by a variety of insults, including cholesterol crystals [73], amyloid-β [74], or cell death [75]. In necrotic cell death, intracellular molecules are released from the cytoplasm of the ruptured cell and stimulate the pattern recognition receptors present on myeloid cells, including microglia. The intracellular molecules act as agonists on the receptors for DAMPs. Prototypical DAMPs include chromatin-associated proteins such as high-mobility group box 1 (HMGB1), heat shock proteins (HSPs), and purine metabolites such as ATP and uric acid [71]. Two principal microglial pathways sensing DAMPS involve TLRs and the nucleotide-binding oligomerization domain-like receptor NLRP3 [71].

Recently, Liddelow and colleagues illuminated the crucial role of microglia in orchestrating brain responses to neuroinflammatory stimuli [76]. The authors demonstrated that lipopolysaccharide (LPS)-activated, neuroinflammatory microglia can induce astrocytes into a neurotoxic, reactive form termed A1. The induction mechanism has been narrowed down to three secreted factors from activated microglia; Il1α, TNFα, and C1q, and these cytokines and complement molecules have been shown to be necessary and sufficient to induce A1 astrocytes. As a consequence, the activated astrocytes secrete a yet-to-be-identified toxin which kills neurons and oligodendrocytes. Habbas et al. studied the roles of TNFα in a different model of inflammation—experimental autoimmune encephalitis (EAE) [77]. They found that inflammatory release of TNFα activates astrocyte TNF receptor type-1 (TNFR1), which in turn triggers an astrocyte-neuron signaling cascade resulting in persistent functional modification of synapses. The authors showed that astrocytic TNFR1 signaling was necessary for the hippocampal synaptic alteration and contextual learning-memory impairment observed in an animal model of multiple sclerosis. Together, these findings underscore the pivotal role of microglia along the microglial-astrocytic-neuronal axis in neurodegenerative diseases and cognitive dysfunctions.

### 4.2. Frequency and Distribution of Ca^2+^ Signals in Neuroinflammatory Microglia

**Figure 2:**
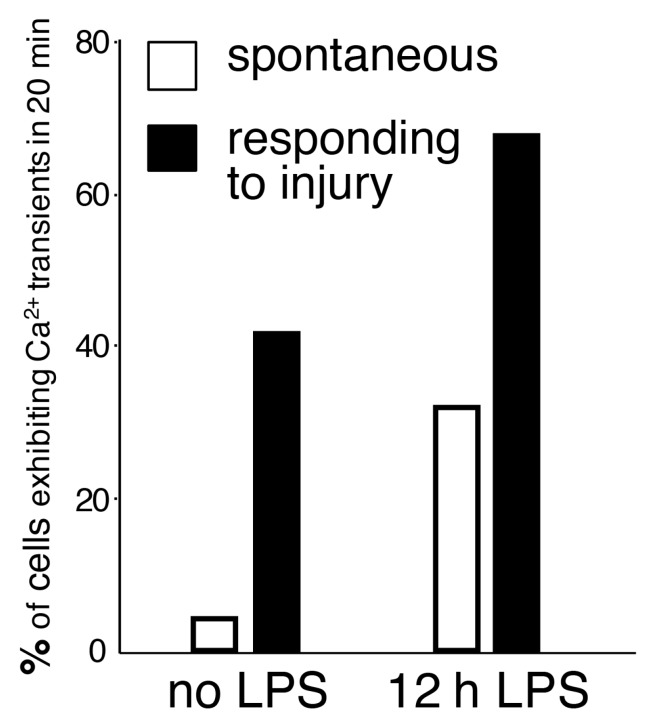
The frequency of spontaneous and evoked Ca^2+^ transients in cortical microglia, calculated as a percentage of cells exhibiting at least one Ca^2+^ spike during a 20-min recording session. Ca^2+^ activity was detected with GCaMP5G and two-photon laser scanning microscopy. In this model, only 4% of resting microglia exhibited spontaneous Ca^2+^ activity (no LPS, white bar). The frequency of Ca^2+^ transients increased 8-fold after LPS exposure (12 h LPS, white bar). In cells extending processes towards the focal laser injury, 67% of microglia displayed Ca^2+^ activity (12 h LPS, black bar). Adapted from [16].

**Figure 3:**
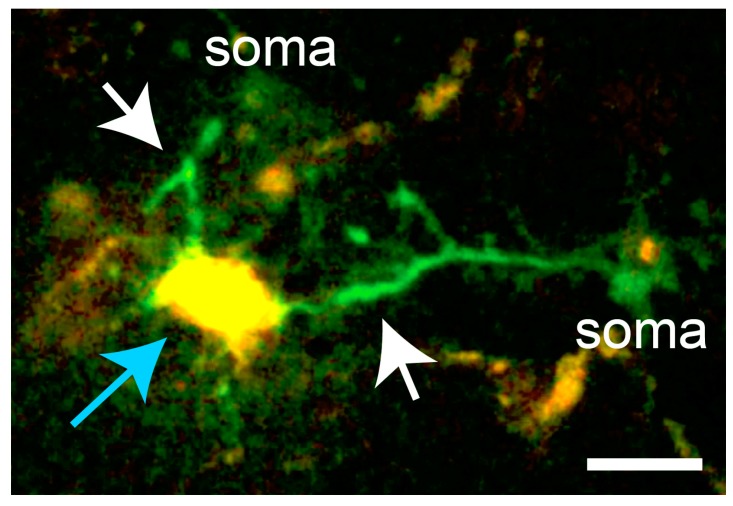
In microglia responding to laser damage (blue arrow), Ca^2+^ transients occur predominantly in the processes extending towards the lesion (white arrows). Scale bar, 10 µm. In vivo imaging data from [16].

While spontaneous microglial Ca^2+^ transients are infrequent, inflammatory stimuli significantly elevate the baseline of spontaneous microglial Ca^2+^ activity. The generation of the PC::G5-tdT reporter mouse enabled us to assess Ca^2+^ activity in resident inflammatory microglia after LPS challenge [16]. LPS was injected subcutaneously in the mandibular lip, as originally described by Lee and collaborators [78]. Subcutaneous LPS administrations result in peripheral inflammation, without blood-brain barrier breach, and subsequent indirect activation of microglia by diffusion of cytokines into the brain parenchyma. Twelve hours after injection, the baseline Ca^2+^ activity was increased 8-fold (Figure 2), escalating the percentage of cells showing any Ca^2+^ transients during a 20-min period to more than 30%. Notably, the majority of injury-induced Ca^2+^ transients (>80%) were localized to the processes (Figure 3), while the remainder occurred both in the processes and cell bodies.

We also applied the paradigm of microglial response to laser burn injury to our Ca^2+^ reporter system. Co-expression of tdTomato and GCaMP5G allowed tracking of the processes extending towards the localized lesion and visualization of Ca^2+^ transients, affording simultaneous measurements of process motility as well as intracellular activity. When these experiments were performed in LPS-primed brains, 67% of microglia responding towards the lesion displayed Ca^2+^ transients (Figure 2).

Remarkably, Ca^2+^ activity considerably decreased at later stages of inflammation, especially after microglia acquired ameboid morphology, typically 24 h after LPS injection. Moreover, Ca^2+^ responses to laser lesions remained low to undetectable for at least one month after a single dose of LPS [16]. This observation is consistent with the view that microglia are capable of retaining long-term memory of infectious insults via a putative epigenetic mechanism which may contribute to chronic inflammation in neurological illnesses [79].

### 4.3. Receptor Pathways and Sources of Microglial Ca^2+^ Transients 

Direct administration of drugs to the brain surface prior to Ca^2+^ in vivo imaging allows initial pharmacological characterization of underlying mechanisms. In our hands, application of pyridoxal-5-phosphate-6-azophenyl-2′,4′-disulfonic acid (PPADS, a non-selective P2 purinergic antagonist) to the dura reduced the frequency of Ca^2+^ transients in responding microglia by >75% [16]. While the general involvement of P2 receptors in mediating Ca^2+^ transients is expected, the relative roles of P2Y and P2X receptor subtypes expressed by microglia (primarily P2ry13, P2ry12, P2ry6, P2rx7, and P2rx4) in modulating Ca^2+^ transients in specific disease states still remains to be characterized in detail. A more complete investigation of microglial Ca^2+^ dynamics will have to include the TRP channels [80,81], adenosine and adrenergic receptors [82,83], and other G-protein coupled receptors [84,85]. Virtually no data is available on the specific roles of TLRs, and cytokine and chemokine receptors in modulating microglial Ca^2+^ signals in vivo. 

Another incompletely clarified issue pertains to the mechanism of Ca^2+^ influx in normal and inflammatory microglia. Similar to other immune cells, microglia robustly express store-operated Ca^2+^ release-activated Ca^2+^ (CRAC) channels, which mediate elevation of cytosolic Ca^2+^ levels after depletion of endoplasmic reticulum Ca^2+^ [27,86]. All three isoforms of the CRAC channel (Orai1, Orai2, and Orai3) are highly expressed by microglia [87], mediating Ca^2+^ influx from the extracellular space. Michaelis et al. explored the role of Orai1 in cultured microglia [88]. However, additional in vivo experiments, including conditional genetic ablations of the corresponding genes, will be required to determine the involvement of specific receptor pathways and CRAC channel isoforms in health and disease. Given that the CRAC channels mediate a broad array of cellular responses, comprising secretion, gene expression, cell growth, and proliferation [89], this research will likely identify new target mechanisms for therapeutic interventions [86].

## 5. Microglial Ca^2+^ Activity in Alzheimer’s Disease and Neurodegeneration

Alzheimer’s disease (AD) is a slowly progressing neurodegenerative disease that is associated with plaque deposits of oligomeric amyloid-β (Aβ) peptides, which are thought to trigger pathological events leading to cognitive decline. Microglia have long been implicated in the etiology of AD because of their dramatic responses to the pathophysiology of the disease. Indeed, recent genome-wide association studies have identified several gene variants selectively expressed in microglia which present an increased risk for the late-onset form of AD, such as *TREM2* or *CD33* [90,91,92]. *TREM2* has been shown to regulate the phagocytic ability of myeloid cells. Certain *TREM2* variants compromise the ability of microglia to internalize Aβ, establishing the phagocytic pathway as one of the key mechanisms in Aβ re-uptake and clearance [93]. In an independent aggravating mechanism, microglia exposed to Aβ engage in excessive synapse pruning in a complement- and CR3 (CD11b/CD18)-dependent fashion, leading, at least in the mouse model, to synapse loss before plaque formation [94]. Hence, in addition to chronic microglial activation, a complementary research focus is also needed on microglia in earlier AD stages when neuronal synapses are already vulnerable to synaptotoxic Aβ oligomers [95].

Chronic microglia-mediated neuroinflammation exacerbates the condition in later stages of the disease. When the ability of microglia to clear amyloid plaques fails, prolonged production of pro-inflammatory cytokines may become detrimental. In a landmark study, Hickman et al. showed by quantitative PCR that in PS1-APP mice, an established mouse model of AD, microglia had a 2.5-fold increase in levels of the proinflammatory cytokines interleukin 1β (IL1β) and tumor necrosis factor TNFα, suggesting that there is an inverse correlation between cytokine production and A**β** clearance [96]. Sustained exposure to A**β**, cytokines, and other inflammatory mediators appear to cause permanent impairment of microglial function at the plaque sites [97]. This dysfunction is manifested by a marked decrease in directed process motility and phagocytic activity in mice with AD-like pathology [98]. New profiling technologies using massively parallel single-cell RNA sequencing will be tremendously helpful in further analyses of microglial disease-associated gene expression [99]. 

However, very few studies looked at the time course of intracellular Ca^2+^ signaling in AD microglia. McLarnon et al. examined cultured microglia isolated from the postmortem brains of AD patients and non-demented controls. They found that AD microglia had significantly higher basal Ca^2+^ and diminished amplitudes of CRAC-mediated Ca^2+^ entry, but prolonged time courses of ATP responses. Overall, these data indicate that significant abnormalities are present in Ca^2+^ signal transduction in AD-patient microglia [100]. In cultured mouse cells, Aβ was shown to trigger increases in intracellular Ca^2+^, ATP release, IL1β secretion, and plasma membrane permeabilization in wild-type microglia, but not in microglia from P2rx7-deleted mice, suggesting that Aβ-mediated activation involves purinergic P2rx7 receptor function [101]. The Garaschuk group investigated Ca^2+^ signaling in mouse AD models in vivo with optimized imaging protocols [102]. In two different mouse models of AD, they showed that plaque-associated microglia failed to respond reliably to extracellular release of ATP [103]. However, these activated microglia had increased incidence of spontaneous Ca^2+^ transients; almost 80% of amoeboid microglia in the plaque vicinity exhibited Ca^2+^ transients. Conversely, the amplitude of Ca^2+^ transients was significantly smaller in amoeboid cells compared to ramified microglia. In ramified microglia located in between amyloid plaques, the amplitude of spontaneous Ca^2+^ transients became significantly larger compared to age-matched controls, but their frequency remained normal. The Ca^2+^ transients in plaque-associated microglia were reversibly inhibited by PPADS, a non-selective antagonist of P2 receptors. Thus, the authors revealed substantial signaling dysfunction in AD microglia, characterized by an increased spontaneous frequency but reduced amplitude of Ca^2+^ signals in plaque-associated amoeboid cells [103]. These results appear to be in general agreement with in vitro data as well as with our analysis of Ca^2+^ activity in LPS-induced neuroinflammatory microglia responding to injury [16]. It is plausible that intracellular hyperactivity in plaque-associated microglia triggers a Ca^2+^-dependent release of proinflammatory cytokines in the vicinity of Aβ deposits and further investigation of this phenomenon is warranted.

Long-term in vivo imaging provided additional novel insights into microglial turnover and migration in mouse AD models. By genetically labeling individual resident microglia, Fuger et al. confirmed that neocortical resident microglia are long-lived with a median lifespan of 15 months, implying that 50% of resident microglia persist the entire mouse lifespan under homeostatic conditions [104]. In the mouse model of AD, however, microglial proliferation was increased threefold. Further analysis indicated that this increase in proliferation occurred in plaque-free areas and the newly emerged cells then moved toward the nearby amyloid plaques [104].

It has been recently demonstrated that microglia-like cells can be generated from human pluripotent stem (iPS) cells or embryonic stem (ES) cells [105,106]. The differentiation protocol yields microglia that are initially amoeboid, proliferating, and capable of extensive migration and robust phagocytosis of CNS substrates. Later, these cells adopt first-order ramified morphologies and when embedded in 3D cultures supplying the neuroglial environment, they project highly branched ramifications. Muffat at al. showed that these microglia-like cells can respond to localized damage in 3D cultures by extending a single long process towards the injury center. Later, they migrate their cell bodies and surround the damaged area, while the microglia farther away from the lesion do not migrate [106]. Interestingly, Abud and collaborators showed that these induced microglia also undergo Ca^2+^ transients and demonstrated that administration of ADP induced Ca^2+^ rises in a P2RY12 receptor-dependent manner [105]. These cells can be also transplanted into transgenic mice and human brain organoids, and resemble microglia in vivo. This new technology opens avenues for the generation and genetic manipulation of human microglia to investigate their function and establish new strategies for therapy of AD and other neurodegenerative conditions.

## 6. Role of Microglial Ca^2+^ Transients in Ischemic Stroke

Microglia are part of the neurovascular unit which comprises brain endothelial cells, pericytes, vascular smooth muscle cells, other glia such as astrocytes and oligodenroglia, and neurons [107]. The neurovascular unit controls the permeability of the blood-brain barrier, cerebral blood flow, and maintains the cerebral fluid homeostasis required for proper functioning of neuronal circuits. Inter-cellular interactions in the neurovascular unit are essential for brain function, and dysfunctional signaling in the neurovascular unit can lead to disease [108]. Aging is a risk factor in neurovascular unit interactions, and is associated with decreased microvessel integrity and increased cerebral tissue susceptibility to ischemic injury and post-ischemic inflammation [109].

Ischemia can develop as a consequence of thrombosis in situ, or following embolic occlusion of a cerebral vessel. Initial events after ischemia result in necrosis of core infarcted tissue and reduced function of adjacent penumbra tissue. After disruption of ATP generation and consequently the function of the Na^+^/K^+^ transporter, cellular depolarization allows Ca^2+^ influx. Further, glutamate accumulation in the extracellular space results in activation of all glutamate receptor subtypes, resulting in excitotoxicity, activation of the intrinsic apoptosis pathway, and cell death [110]. Evolution of ischemic injury and cell death continues for minutes, hours, and even days, depending on the vulnerability of the particular brain region [108].

In addition to excitotoxicity at the cellular level, the release of glutamate and ionic imbalance impact ischemic injury progression at the tissue level. They trigger cortical spreading depolarization (CSD), a transient and slowly propagating (2–5 mm per minute) wave of near-complete depolarization of neurons and astrocytes. This is followed by a period of electrical depression, associated with major transmembrane ionic and water shifts. This phenomenon is highly evolutionarily conserved among vertebrates and invertebrates [111]. It has been detected in clinical cases of stroke and traumatic injury, and it is also associated with migraine auras [112]. Spreading depolarizations accelerate tissue damage during brain injury by inducing neuronal death in energy-compromised tissue. Further, CSD waves can propagate from energy-depleted tissue into surrounding, well-nourished tissue, as is often the case in stroke and brain trauma. Hence, CSD suppression may prove worthwhile for reducing infarct maturation [108,112,113,114,115].

**Figure 4:**
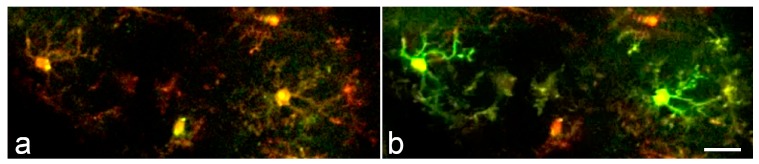
Synchronous microglial Ca^2+^ activity in the mouse cortex. (**a**,**b**) Time lapse two-photon images acquired within a 20-second interval from a LPS- and bicuculline-treated animal demonstrate synchronized microglial Ca^2+^ transients. Scale bar: 20 μm. Modified from [16].

Cerebral ischemia also elicits robust neuroinflammatory responses which involve activation of microglia and other immune cells. Microglia have a complex role in stroke pathophysiology because they orchestrate both neuroinflammatory and neuroprotective responses, and much research is focused on finding the optimal balance between the inflammatory and neurotrophic phenotypes [116,117]. Szalay and colleagues have investigated the effect of selective ablation of microglia during acute ischemic stroke following transient middle cerebral artery occlusion (MCAo) [118]. They found that a near-complete ablation of microglia with PLX3397, an inhibitor of the Csf1r pathway, resulted in a 60% increase in infarct size following MCAo. This effect was reversed when microglia were allowed to repopulate the brain prior to the ischemic injury. The absence of microglia also disrupted Ca^2+^ signaling in neurons and increased neuronal death. Remarkably, microglial ablation also significantly reduced the incidence of cortical spreading depolarizations induced by MCAo [118]. These results underscore the importance of microglia in neuronal network activity and confirm their role in the CSD phenomena [119]. As yet, there is no published information available regarding whether microglial Ca^2+^ signaling is affected by CSD. Mouse reporters harboring pan-microglial GECI indicators will afford a distinct advantage in addressing this question. Indeed, we have previously shown that detecting synchronous waves of Ca^2+^ activity is feasible with the PC::G5-tdT reporter (Figure 4) [16], and work is currently underway to characterize microglial Ca^2+^ signaling following MCAo. It is noteworthy that in hemorrhagic strokes, not covered here in detail, new research indicates that subarachnoid blood per sig may not be sufficient to trigger a CSD in rodents [120]. However, subarachnoid infusions of fresh blood are sufficient to cause clusters of spreading depolarizations in the gyrencephalic swine brain [121]. In both models, reciprocal interactions between focal cerebral ischemia and subarachnoid hemorrhage appear to synergize to facilitate recurrent CSDs, leading to delayed cerebral ischemia syndrome [120,121].

It has been shown that CSD stimulates microglial secretion of Il1β [122] and TNFα [123]. TNFα lowers the threshold for CSD induction, promoting perpetuation of CSD induction through positive feedback [123,124]. Conversely, TNFα can also reduce CSD amplitude in the rat cortex [125]. Insulin-like growth factor IGF1, a neurotrophic factor secreted by microglia, abrogates TNFα induction by CSD [123], and so does environmental enrichment which promotes neuroprotective phenotypes in microglia [126]. Evidently, the effects of inflammatory cytokines in stroke progression are significant and complex, and uncovering the patterns and potential blockers of Ca^2+^ activity in microglia may instruct new ways to control cytokine release. Currently, anti-inflammatory mechanisms are being evaluated in a clinical phase II trial of ischemic stroke which targets the interleukin-1 signaling pathway via recombinant antagonist IL-1Ra/anakinra [127]. It is plausible to suggest that better understanding microglial Ca^2+^ dynamics in the course of ischemic injury and spreading depolarizations will identify new targets for therapeutic intervention.

## 7. Future Directions

Future progress in this research field will benefit from improved animal reporters capable of multiplex data acquisition, enabling new ways to study specific cell-cell interactions. There is a need for genetic tools that will allow simultaneous imaging of activity in microglia and other glial cells such as astrocytes, using, for instance, the newly developed red-shifted indicators along with green light-emitting tools. Systems allowing parallel recordings of glutamate and Ca^2+^ concentrations are also very desirable, especially in combination with long-term imaging in awake animals. Furthermore, expanding the toolbox of fluorescently tagged reporters of key immune effectors will facilitate deeper mechanistic insights into mouse models of neuroinflammation and neurodegenerative diseases. 

## Abbreviations

AAV	adeno-associated virus
Aβ	amyloid beta
AD	Alzheimer’s Disease
ADP	adenosine diphosphate
ATP	adenosine triphosphate
CRAC	Ca^2+^ release activated Ca^2+^ current
CSD	cortical spreading depression
DAMP	damage-associated molecular patterns
ER	estrogen receptor
FRET	fluorescence (Förster) resonance energy transfer
GECI	genetically encoded Ca^2+^ indicator
GFP	green fluorescent protein
LPS	lipopolysaccharide
MCAo	middle cerebral artery occlusion
miR-9	microRNA-9
PAMP	pathogen-associated molecular patterns
PMT	photomultiplier tube
PPADS	pyridoxal-5-phosphate-6-azophenyl-2′,4′-disulfonic acid
RFP	red fluorescent protein
TLR	Toll-like receptor
